# Meta-neural-network for real-time and passive deep-learning-based object recognition

**DOI:** 10.1038/s41467-020-19693-x

**Published:** 2020-12-09

**Authors:** Jingkai Weng, Yujiang Ding, Chengbo Hu, Xue-Feng Zhu, Bin Liang, Jing Yang, Jianchun Cheng

**Affiliations:** 1 Key Laboratory of Modern Acoustics, MOE, Institute of Acoustics, Department of Physics, Collaborative Innovation Center of Advanced Microstructures, Nanjing University, 210093 Nanjing, P. R. China; 2 School of Physics and Innovation Institute, Huazhong University of Science and Technology, 430074 Wuhan, Hubei P. R. China

**Keywords:** Computational science, Acoustics

## Abstract

Analyzing scattered wave to recognize object is of fundamental significance in wave physics. Recently-emerged deep learning technique achieved great success in interpreting wave field such as in ultrasound non-destructive testing and disease diagnosis, but conventionally need time-consuming computer postprocessing or bulky-sized diffractive elements. Here we theoretically propose and experimentally demonstrate a purely-passive and small-footprint meta-neural-network for real-time recognizing complicated objects by analyzing acoustic scattering. We prove meta-neural-network mimics a standard neural network despite its compactness, thanks to unique capability of its metamaterial unit-cells (dubbed meta-neurons) to produce deep-subwavelength phase shift as training parameters. The resulting device exhibits the “intelligence” to perform desired tasks with potential to overcome the current limitations, showcased by two distinctive examples of handwritten digit recognition and discerning misaligned orbital-angular-momentum vortices. Our mechanism opens the route to new metamaterial-based deep-learning paradigms and enable conceptual devices automatically analyzing signals, with far-reaching implications for acoustics and related fields.

## Abstract (web summary)

The authors present a passive meta-neural-network for real-time recognition of objects by analysis of acoustic scattering. It consists of unit cells termed meta-neurons, mimicking an analogous neural network for classical waves, and is shown to recognise handwritten digits and misaligned orbital-angular-momentum vortices.

## Introduction

It is a fundamental problem in wave physics to detect and recognize the geometric shapes of objects by properly analyzing the scattered wave, representing the most basic challenge behind a plethora of important applications. Representatively, in acoustics, typical examples range from medical ultrasound imaging^1^ to industrial non-destructive evaluation^2^ to underwater detection^3^. In contrast to the conventional mechanisms that rely on human experts such as physicians interpreting the medical ultrasonic images in the clinic, which would inevitably suffer from low efficiency, potential fatigue and wide variations in pathology^4–6^, the recent emergence of computer-assisted deep-learning techniques^7^ has achieved state-of-the-art performance in the important problem of identification and classification of medical images of scattered acoustic fields such as for detection of anatomical structures and disease diagnosis and so on^6,8,9^, among other fascinating applications in speech recognition^10–12^, emotion analysis^13–16^, etc. In spite of the remarkable improvement in performance and simplification in process, however, such a shift of the burden from human to computers would still arouse the issue of computational complexity, energy supply, device size and cost, owing to their dependence on precise acoustic images that need to be measured via sensor-scanning and computer-based postprocessing. It is therefore essential to continuously pursue new deep-learning-based mechanisms with simpler design, smaller footprint, faster speed and reduced energy consumption and fewer sensors, which would be vital for the real-world application in many diverse scenarios such as medical imaging where fast and easy assessment of tissues are highly desired.

In this article, we break through such fundamental barriers by introducing a physical mechanism to use a passive meta-neural-network comprising a three-dimensional matrix of metamaterial unit cells, with each serving as a meta-neuron, to mimic an analogous neural network for classical waves with compactness, simplicity, and pure-hardware task-solving capability. The recent rapid expansion of the research fields of photonic/phononic crystals^17–22^ and metamaterials^23,24^ enables unconventional manipulation of wave fields, such as anomalous refraction/reflection^25,26^, invisibility^27,28^, rectification^29,30^ etc., in a deterministic manner, relying on rational design based on human knowledge. The past few years witness considerable efforts devoted to applying machine learning in these artificial structures, but merely aiming at designs of active imaging devices with reduced complexity^31^ or metamaterials for producing specific wave fields^32–35^. Recently passive neural networks are proven possible by using diffractive layers with locally-modulated thickness according to machine-learning training results^36^, which generates quasi-continuous phase profiles and results in significant phase variation only over wavelength-scale distance^37^. Besides, optical metamaterial-based neural network is theoretically proposed with metasurfaces^38^ or nanostructured medium^39^. In contrast, here we present a theoretical and experimental work of endowing passive acoustic metamaterials with the “intelligence” to perform complex machine-learning tasks. We prove that metamaterials’ extraordinary capability to provide abrupt phase shift within deep-subwavelength scales in all three dimensions is pivotal for the equivalence between conventional and the proposed neural network, and use a computer to train the designed meta-neural-network by iteratively adjusting the whole phase profile of each layer of meta-neurons. The resulting meta-neural-network features planar profile, high spatial density of meta-neurons, and subwavelength thickness of each meta-neural-layer, which are particularly crucial for acoustic waves that generally have macroscopic wavelength. More importantly, We experimentally demonstrate a compact passive metamaterial-based neural network capable of directly recognizing complex objects in real 3D space in a totally passive, real-time, sensor-scanning-free and postprocessing-free manner, as will be demonstrated hereafter.

## Results

### Theory of meta-neural-network

**Fig. 1:**
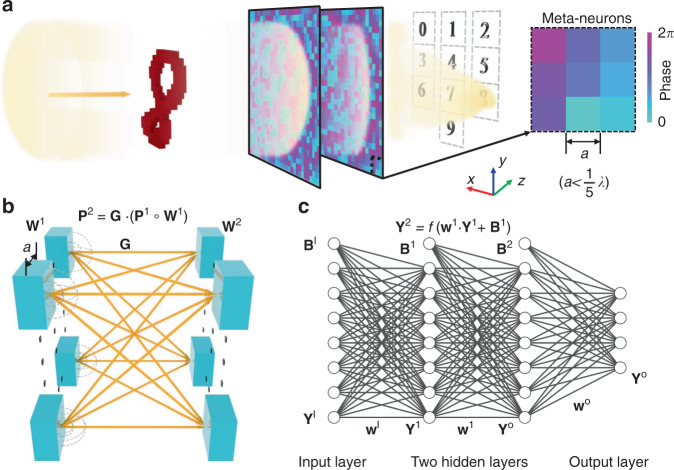
Passive object recognition by acoustic meta-neural-network. **a** The proposed meta-neural-network with network parameters given by a computer-aided training process is capable of converging the scattered energy from the object (chosen as handwritten digit “8” here) into the corresponding region on the detection plane (marked by dot-line boxes behind the last layer). **b** Schematically illustrates the interaction between two adjacent 2D layers of meta-neurons whose deep-subwavelength size physically ensures wave propagation from each meta-neuron on the 1st layer to all meta-neurons on the 2nd one (after undergoing the phase-amplitude modulation by the 1st layer and free-space diffraction in between, described by **W**^1^ and **G**, respectively). **c** A conventional neural network can be accurately mimicked by the practical physics model shown in **b** even for compact device and/or complicated object.

Figure 1 schematically shows our proposed mechanism of constructing an acoustic meta-neural-network comprising multiple parallel layers of subwavelength meta-neurons for passive and real-time recognition and classification of objects by the geometric shape. The object to be examined is illuminated normally by a monochromatic plane wave, and the meta-neural-network is located at the transmitted side to receive the scattered acoustic wave produced by the object. The key role of the meta-neural-network is to interact with the incident wave after it is rebounded by the object and thereby converges the acoustic energy, which would scatter into all different directions in its absence, to the desired region on a detection plane behind the last layer, as illustrated in Fig. 1a. For explaining the recognition criterion of meta-neural-network, we exemplify the detection plane for a typical case where 10 handwritten digits, from 0 to 9, are chosen as the object for recognition. The detection plane includes 10 identical square regions assigned respectively for these 10 objects. For a specific object, only when the output signal eventually yielded by the meta-neural-network is accurately redistributed on the detection plane such that the total intensity in the expected region corresponding to this digit is higher than the rest regions, can the recognition and classification be considered successful. For better mimicking the real-world applications, here we do not directly translate the image recognition mechanism for visible light to acoustics by simply using the image of digits as the input pattern or vectorizing the input images for facilitating 2D on-chip applications and, instead, attempt to realize real-time and high-accuracy recognition of object by appropriately analyzing its scattered wave field.

First, we consider the propagation of scattered wave in such a multi-layered metamaterial system. As the fundamental building block of our designed meta-neuron-network, each meta-neuron modulates the amplitude and phase of the incident wave, then the outgoing wave on the transmitted side serves as second sources and becomes the input signal for the next layer, as governed by Huygens’ principle^40^. Obviously, the radiation pattern of each meta-neuron depends on the unit cell size and spacing related to wavelength. When each meta-neuron can be approximated as a monopole source, the relationship between the wave fields on two neighboring layers in our meta-neural-network can be written as

1 $${\mathbf{P}}^{l + 1} = {\mathbf{G}}^l \cdot ({\mathbf{P}}^l \circ {\mathbf{W}}^l),$$

where vector **P**^*l*+1^ denotes the input wave of the (*l*+1)-th layer of meta-neurons, **G**^*l*^ is the wave propagation matrix (see the Supplementary Notes 1 and 2), ${\mathbf{W}}^l = {\mathbf{t}}^l{\mathrm{exp}}({\mathrm{j}}{\mathbf{\varphi }}^l)$ is the modulation introduced by the meta-neurons on the *l*-th layer with **t**^*l*^ and **φ**^*l*^ referring to the amplitude and phase modulation respectively, “$\circ$” denotes the element-wise multiplication. While the conventional neural network can be written as

2 $${\mathbf{Y}}^{l + 1} = f({\mathbf{w}}^l \cdot {\mathbf{Y}}^l + {\mathbf{B}}^l),$$

where *f* is the nonlinear activation function, **w**^*l*^ is the weight and **B**^*l*^ is the bias.

Comparison of Eqs. (1) and (2) clearly reveals the equivalence and differences between meta-neural-network and a conventional neural network. Unlike the characteristic of the weight as the learnable parameters in conventional neural networks, the wave propagation function is fixed once the meta-neural-network is fabricated which determines the axis distance between the adjacent layers. This suggests that the wave propagation function, which forms the connections between the adjacent layers, is more like a hyperparameter than a learnable parameter, and it is not necessary to optimize the axis distance during the training process in the design of meta-neural-network. The wave propagation function also prevents the multi-layered meta-neural-network from degenerating into a monolayer meta-neural-network in physical systems, rather than merely forming the connection between adjacent layers (see Supplementary Notes 3 for details). In the conventional neural network, the “weights” represents the connecting strength between two neurons in adjacent layers, and the input of the latter layer is determined by the output values of the former layer and the ‘weights’ between them. By tuning the weights, the output loss is continuously decreasing, and finally the neural-network will be capable of accomplishing specific tasks. Similarly, the learnable parameters in our meta-neural-network are the phase modulation provided by the meta-neurons. The input of meta-neurons in the latter layer is the interference of outgoing wave emitted by the whole neurons in the former layer. And the adjusting of phase modulation redistributes the wave energy on the output plane, leading to continuous decrease of the loss and the functionality of resulting meta-neural-network to perform tasks in the same way as the conventional neural network. (See Supplementary Note 2 for details).

It is apparent, however, that such equivalence between the mathematical model and practical physical system requires effective connection between each meta-neuron and all the meta-neurons on the neighboring layer, which would be difficult for bulky diffractive components modulating phase continuously when the system has a compact size or the object has a complicated pattern. In contrast, meta-neurons’ unique capability of metamaterials to offer arbitrary and abrupt phase shift^41–46^ validates the monopole approximation required by Eq. (1) which is the hinge of the physical analogy of a standard neural network(see Supplementary Note 1 for details). Given that the transmission loss of meta-neurons is trivial, the phase modulation essentially plays the same role as the weight in conventional deep-neural-network, and we, therefore, choose phase shifts of meta-neurons as the learnable parameters for training as will be shown later.

Notice that the proposed strategy needs no measurement of the original scattered field nor reconstruction of the precise acoustic image, exempted from the burden on the cost and time in conventional computer-assisted deep-learning paradigms which will further increase when the object complexity is enhanced or the detection region is enlarged. Limited by the current technology, this will result in many challenges including implementing large-scaled phased arrays^47^, fabricating subwavelength sensor (e.g. piezoelectric transducer), and accelerating measurements and analysis of huge amount of sound field data. In stark contrast, the meta-neural-network performs detection and computation simultaneously due to the parallel interaction between wave and meta-neurons without sensor-scanning or postprocessing, which accomplishes once the incident wave passes regardless of the resolution or number of meta-neurons, and the output field only needs to be measured at the receiving end with fixed number of sensors (e.g., Fig. 1a) as few as the possible classification types of objects, no matter how complicated the target is. In addition to these advantages of passive elements in terms of speed and simplicity, our proposed meta-neural-network with compact planar geometry and ultra-fine phase resolution enables downsizing the device to the scale unattainable with diffractive components and recognizing objects excessively complicated for diffractive neural networks, as we will demonstrate in what follows (see Supplementary Note 4 for details).

### Experimental realization of handwritten digits classification

**Fig. 2:**
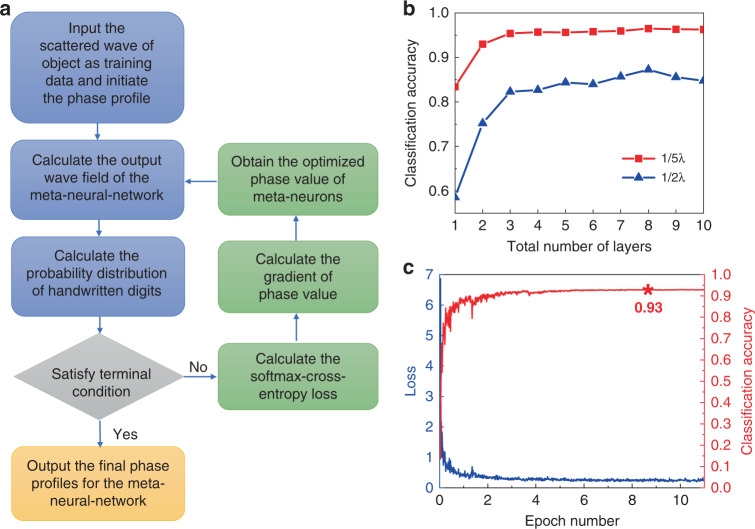
Simulated results for the meta-neural-network. **a** The chart flow of the training process that uses the scattered wave produced by different objects as training data and calculates the loss of meta-neural-work to iteratively tune the phase value of each meta-neuron, until achieving the maximal probability of converging the scattered energy produced by a specific class of objects into the predesigned region. **b** Shows the comparison between the simulated classification accuracy as function of the total layer number for meta-neural-networks with different meta-neuron size. **c** Depicts the simulated dependence of loss value and classification accuracy on the epoch number, showing that the accuracy increases with epoch number and eventually reaches the maximum (93%) in the training process of our designed meta-neural-network.

To manifest the unique advantages of our proposed meta-neural-networks in terms of compactness and efficiency, we first choose to demonstrate via both simulation and experiment the recognition of MNIST (Modified National Institute of Standards and Technology) handwritten digits on a scale approximately one order of magnitude smaller than attainable with deep-learning-based diffractive layers. The database contains 55,000 training images, 5000 validation images, and 10000 testing images. For simplifying the design and fabrication of meta-neural-network sample in the following experiments, we avoid simultaneous adjustment of amplitude and phase for the transmitted wave and only use phase modulation with the transmission efficiency being set to be 1, which does not appreciably affect the accuracy of the resulting device as we demonstrate via numerical simulation (see the Supplementary Notes 5 and 6). Each object is implemented based on a binary image formed by rounding up the grayscale value of each pixel in the corresponding MNIST image (see Supplementary Note 7). The details of training process are shown in Fig. 2a. The softmax-cross-entropy loss function^48^ which is commonly used in classification problem is introduced (see detailed discussions in Supplementary Note 2), and the gradient of phase value is calculated through error back-propagation algorithm^49^. We adjust the phase values of meta-neurons in search of the minimum of loss value corresponding to the maximum likelihood of making the total acoustic intensity in the target region higher than the others for as many digits as possible in the MNIST database. By iteratively feeding training data, the classification accuracy of testing data keeps increasing and eventually becomes stable within 6 epochs.

In our simulation, the operating frequency is set to be 3 kHz (corresponding to a wavelength of ~11.4 cm in air) such that the experimental sample of meta-neural-network is of moderate size which facilitates both the 3D printing fabrication of subwavelength meta-neurons and the sound field measurement in anechoic chamber. As a specific design, each layer is chosen to consist of 28 × 28 (784 in total) meta-neurons, equal to the number of pixels in a handwritten digits picture in the MNIST database. Each individual meta-neuron is assumed to have a sub-wavelength size in each dimension, consistent with the actual size of the practical metamaterial we will implement in the measurement. Specifically, the transversal size of the meta-neuron is 2 cm (smaller than 1/5 wavelength), which helps to ensure deep-subwavelength resolution of meta-neural-network that is vital for the high-accuracy recognition for more sophisticated cases. The axial distance between two neighboring layers is set to be 17.5 cm. After its training, the design of our meta-neuron digit classifier is numerically tested by 10,000 images from MNIST testing dataset.

Here we choose a design of meta-neural-network consisting of two layers of metamaterial only for a balance between the classification accuracy and efficiency, based on our numerical analysis on the dependence of accuracy on the layer number as shown in Fig. 2b which indicates that the increase rate of accuracy with respect to layer number becomes much slower for designs containing more than two layers. The accuracy of recognition by such a simple bilayer structure can reach 93%, which is considerably high given the significant acceleration of training process, reduction of meta-neuron number and downscaling of resulting device, and can be further improved at the cost of increasing the total number of meta-neurons and enhancing the fabrication precision of unit cells as implied by observing Fig. 2b. For comparison, we also calculate the recognition accuracy when each basic building block becomes one-half wavelength wide and the layer distance is chosen such that the equivlance in Eq. (1) holds and plot the numerical results in Fig. 2b which clearly show that the increase of unit size leads to notable deterioration of the performance of meta-neural-network.

Next, we perform experimental measurements to verify our proposed mechanism. As a practical implementation, in the current study, we propose to design a metamaterial unit cell composed of four local resonators and a straight pipe^50^, as illustrated in Supplementary Fig. 6. Such a specific design enables free control of the propagation phase within the full 0-to-2π range while keeping high transmission efficiency via adjustment of a single structural parameter *h*, as shown in Supplementary Fig. 6. Hence the meta-neuron layer has planar profile, subwavelength thickness and, in particular, fine phase resolution (~1/5 wavelength) pivotal for ensuring equivalence between the standard and our metamaterial-based neural network (see Supplementary Notes 1, 2, and 5 for details). Based on the parameter dependence of phase shift given by the numerical simulation, we determined the precise geometric parameter for each meta-neuron and fabricated a meta-neural-network comprising two layers with transversal size of 56 × 56 cm^2^.

**Fig. 3:**
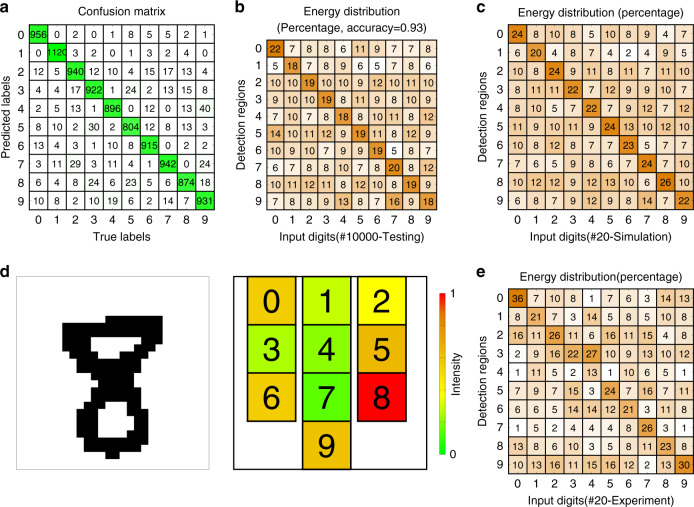
Experimental verification of acoustic meta-neural-network. **a** Shows the confusion matrix for the numerical results of two-layers meta-neural-network with 10,000 handwritten digits. **b** The energy distribution percentage of 10000 handwritten digits. **c** Shows the energy distribution of 20 selected digits in simulation. **d** Total acoustic intensity measured in each detection region corresponding to digits “8”. **e** Is the same as **c** but for the experiments.

With our designed meta-neural-network, the handwritten digits in the testing dataset have been well classified which corresponds to an appropriate redistribution of acoustic energy into the target region, as shown in Fig. 3a, b. In the experiment, we have fabricated 2 sets of steel plates with shapes of handwritten digits (viz., 20 objects in total, and the simulation result is shown in Fig. 3c) which are selected from the testing images that have been numerically proven capable of being correctly classified by our designed meta-neural-network with each meta-neuron endowed with the ideal phase value given by the computer-aided training process. Good agreement is observed between the theoretical and experimental results as shown in Fig. 3d which takes the digit “8” as an example (more details and results in Supplementary Note 8), with both revealing that our designed double-layered meta-neural-network accurately redistributes the input energy into the detection region assigned to the object, except for the poor performance of meta-neural-network when recognizing digit “4” which primarily stems from the experimental error (see the Fig. 3e and Supplementary Note 9).

### The recognition of multiplexed OAM beams

**Fig. 4:**
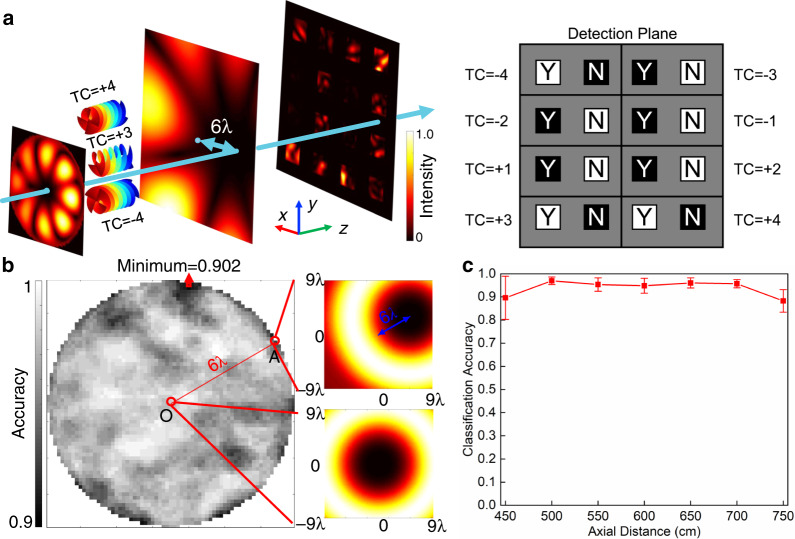
The recognition of misaligned OAM states. **a** Shows the recognition of a multiplexed OAM beam (with TCs = +3, ±4 and a misalignment distance of 6*λ* as an example) by the designed meta-neural-network that redistributes the incident energy on the detection plane in a way such that whether or not each OAM state can be unambiguously marked. **b** Depicts the dependence of recognition accuracy on the distance and direction of misalignment. Insets: the spatial patterns of a misaligned (top) and an aligned (bottom) OAM beams with the same OAM order. **c** Shows the simulated recognition accuracy as a function of axial distance, and the error bar indicates the ±1 standard deviation from the mean of accuracies.

For further demonstrating the potential of our meta-neural-network to recognize very complicated object in real-time with compact footprint, we showcase a distinctive example in which one needs to accurately distinguish between different spatial patterns of wave field that are encoded with information and far more sophisticated than the scattered patterns produced by simple digit-shaped objects. As a representative case, the introduction of orbital angular momentum (OAM) opens a new degree of freedom for information encoding and dramatically improves the capacity of waves as information carriers^51,52^, which is of crucial significance particularly for acoustic waves that dominate underwater communications but innately bear no spin^53–55^. Such spatial multiplexing mechanism uses several twisted beams with different topological charges (TCs) to carry multiplexed information which, however, needs to be read out accurately from the complicated spatial pattern of this synthesized beam. But the existing strategies based on OAM’s orthogonality for passive decoding suffer from uncontrollable spatial locations of the different output beam and, in particular, strict alignment between the beam and receiving device which is vital for decoding accuracy but challenging in practice^56,57^. Here we propose to overcome these fundamental limitations based on an inherently different mechanism, by using an acoustic meta-neural-network trained to recognize the complicated spatial patterns associated with different OAM orders. More importantly, by straightforwardly training the meta-neural-network with both centered and non-centered OAM beams, the system is able to recognize the spatial pattern of each OAM order regardless of whether the centers of the beam and device are perfectly overlapped. A four-layered meta-neural-network containing 101 × 101 × 4 (40,804 in total) meta-neurons is designed to recognize a maximal combination of 8 OAM orders (±1, ±2, ±3, ±4, 255 combinations in total). In the current design, we will demonstrate the realization of a meta-neural-network capable of recognizing multiple OAM beams with their centers transversally misaligned in arbitrary directions by a maximal distance of 6*λ*, which reaches 1/3 of the side length of each meta-neuron layer and would be quite challenging for existing mechanisms using equal-sized devices. The ranges of *r* and *θ* are [0,6*λ*] and [0,2π), respectively, with (*r*,*θ*) being the location of the vortex center under polar coordinate. Figure 4a shows schematically how the designed meta-neural-network realizes accurate and real-time recognition of each OAM beam via elaborate redistribution of the incident energy on the detection plane (which illustrates the recognition of OAM beams composed of +3 and 4 orders with misalignment of (6*λ, *0) as an example). Now the detection plane is divided into 8 regions, with each containing two areas (marked by “Y” and “N”, corresponding to existence and non-existence of a specific OAM state respectively), as shown in Fig. 4a (more details in Supplementary Note 10). The distribution of sound intensity at detection plane is also shown in Fig. 4a, which clearly indicates that the sound energy is redistributed into correct area (more details in Supplementary Note 10). Figure 4b illustrates the dependence of recognition accuracy on the distance and direction of misalignment (viz., the parameters of *r* and *θ*). The significant misalignment can be observed from the comparison between the spatial patterns depicted in the insets for an aligned and misaligned OAM beam with the same order. We have calculated the recognition accuracy for all the possible 255 combinations among 8 orders of OAM states under different (*r*,*θ*) and plot the results in Fig. 4b, which clearly reveals that our mechanism is effective even the distance between the centers of OAM beams and meta-neuron layer reaches 6*λ*. In the training process, we have also taken the parameter of propagation distance of OAM beams into account, in an attempt to also empower the designed meta-neural-network with high tolerance against misalignment of the detection device along the propagation direction which would be of great importance for the practical application of OAM-based communication. The simulated recognition accuracy as a function of axis distance depicted in Fig. 4c shows a high accuracy of our meta-neural-network persisting within a wide range of propagation distance (from 500 cm to 700 cm, nearly 18*λ*). As a result of such distinctive mechanism, we realize real-time and passive recognition of each mutually-orthogonal OAM states by using meta-neural-network that features controllable output regions and high robustness against misalignment along both the axial and transverse directions, which helps to solve the long-standing questions in OAM-based high-capacity communications and would have far-reaching implication in relevant fields by serving as a smart transducer, with the potential to be extended for recognizing more complicated objects given sufficiently-large training database and accordingly-redesigned meta-neurons, e.g., diagnosing tumors in ultrasound imaging or identifying defects in industrial testing.

## Discussion

For clear demonstration of physical model and facilitation of practical implementation, we only demonstrate a considerably reduced model of meta-neural-network, with several major simplifications which, however, will not impair the generality of our proposed mechanism. To be specific, the holistic performance of the current meta-neural-network can be further improved by modifying the design and training of meta-neurons. For example, one can easily enhance its compactness and efficiency by replacing the simple metamaterial unit cell used here with some recently-emerged designs such as hollow-out-type metamaterial with thinner than 1/600 wavelength^46^, and allows programmable meta-neural-network by using reconfigurable meta-neurons. Our scheme also applies to more realistic applications such as ultrasound imaging by employing waterborne metamaterials such as with soft graded-porous media^58^ and including non-planar incident wave and inhomogeneous medium in the training process.

In conclusion, we demonstrate a theoretical design and experimental implementation of a metamaterial-based passive neural network in acoustics, performing various complicated object recognition tasks such as recognition of handwritten digits and misaligned OAM beams. Besides having no dependence on human experts as in computer-based deep-learning methods, our proposed meta-neural-network needs no complicated sensor arrays nor high-cost computers, and, in particular, performs real-time recognition without power supply, thanks to its passive nature and parallel wave-interaction, exempt from the heavy burden on the computational hardware in conventional deep-learning methods. Furthermore, the meta-neural-networks have small footprint thanks to the subwavelength nature of metamaterials, which is vital for their application in acoustics where acoustic waves generally have macroscopic wavelength but unachievable with diffractive components-based neural networks. Our design with simplicity, compactness, and efficiency offers the possibility of miniaturization and integration of deep-learning devices, and may even open route to the design of new generation of conceptual acoustic devices such as portable and smart transducers which, as a result of coupling the functionalities of detection and computation, may be able to automatically analyze the backscattered acoustic signals it receives and subsequently complete sophisticated tasks such as evaluating tumors in a totally passive, sensor-scanning-free and postprocessing-free manner. Furthermore, our designed device serves as a new class of passive deep-learning chips for power-supply-free yet real-time task-solving purpose, with the ability to inspire relevant researches for other classical waves.

## Methods

Our acoustic meta-neural network was simulated using MATLAB and trained in a desktop with a GeForce RTX 2070 Graphical Processing Unit(GPU), Intel(R) Xeon(R) CPU E5-2620 v3 @ 2.40 GHz and 160 GB of RAM, running Windows 7 operating system(Microsoft).

In the experiment, the input sound was generated by a speaker (Beyma CP380), driven by the waveform generator (RIGOL DG1022). The sensor we used on the detection plane was 1/4-inch free field microphone (BRÜEL & KJÆR Type 4961) and the stand-alone recorder (BRÜEL & KJÆR Type 3160-A-022). The experiments are carried out in anechoic room.

## Supplementary information

Supplementary Information

## Data Availability

The data that support the findings of this study are available within the paper and the Supplementary Information. Additional data related to this paper are available from the corresponding authors upon reasonable request. Source data are provided with this paper.

## Source data

Source Data

## Code availability

The code that supports the findings of this study are available from the corresponding author upon reasonable request.
