# Supplementary material for: Meta-neural-network for real-time and passive deep-learning-based object recognition
Source: Nat Commun. 2020 Dec 9;11:6309. doi: 10.1038/s41467-020-19693-x (PMC7725829; doi:10.1038/s41467-020-19693-x)
Supplement: Supplementary file 1 — Supplementary Information [file 41467_2020_19693_MOESM1_ESM.pdf]

Supplementary Information for

Meta-neural-network for Real-time and Passive Deep-learning-based Object Recognition

Jingkai Weng[1*], Yujiang Ding[1*], Chengbo Hu[1], Xue-Feng Zhu[2], Bin Liang[1†], Jing Yang[1] and Jianchun Cheng[1†]

[1]*Key Laboratory of Modern Acoustics, MOE, Institute of Acoustics, Department of Physics, Collaborative Innovation Center of Advanced Microstructures, Nanjing University, Nanjing* 210093*, P. R. China*

[2]*School of Physics and Innovation Institute, Huazhong University of Science and Technology, Wuhan, Hubei 430074, P. R. China*

[*]These two authors contributed equally to this work.

[†]Correspondence and requests for materials should be addressed to B.L. (email: liangbin@nju.edu.cn) or to J.C. (email: jccheng@nju.edu.cn)

Supplementary Note 1. Monopole source approximation

Owing to airborne sound-hard walls in metamaterials, these deep subwavelength meta-neurons could be regarded as acoustic waveguides with specific phase modulation. The sound-hard material is thick enough to avoid wave coupling between adjacent unit cells[1], which is totally different from the diffractive elements. Based on the Huygens-Fresnel principle, every point of a wavefront can be considered as a center of a secondary disturbance which gives rise to spherical wavelets, and the wavefront at any later instant can be regarded as the envelope of these wavelets[2]. Due to its finite geometric size, each meta-neuron can be treated as a square source on the plane incident wavefront with the amplitude t^l and phase φ^l in the progress of wave propagation. Then the acoustic pressure at an arbitrary location in the next layer can be calculated by using the Helmholtz–Kirchhoff formula, as follows

$$P(\mathbf{r}') = \iint_{Sq} \frac{e^{-\mathrm{j}k|\mathbf{r}-\mathbf{r}'|}}{2\pi|\mathbf{r}-\mathbf{r}'|}\left(\mathrm{j}k + \frac{1}{|\mathbf{r}-\mathbf{r}'|}\right)\frac{z-z'}{|\mathbf{r}-\mathbf{r}'|} t^l e^{\mathrm{j}\varphi^l} \mathrm{d}S, \quad (1)$$

where $\mathbf{r} = (x, y, z)$ and $\mathbf{r}' = (x', y', z')$ refer to the source and observation points on two adjacent layers, respectively. Since the exact evaluation of the total field is difficult and requires solutions of the boundary, for the purpose of simplifying our meta-neuron network we make the following assumptions $k|\mathbf{r}-\mathbf{r}'| \gg 1$, $\frac{1}{|\mathbf{r}-\mathbf{r}'|} \approx \frac{1}{r_0}$, $\frac{z-z'}{|\mathbf{r}-\mathbf{r}'|} = \cos\alpha$ and $|\mathbf{r}-\mathbf{r}'| = [(z-z')^2 + (x-x')^2 + (y-y')^2]^{\frac{1}{2}} \approx z' - z + \frac{x'^2+y'^2}{2(z'-z)} - \frac{xx'+yy'}{z'-z} + \frac{x^2+y^2}{2(z'-z)}$, where α is the azimuthal angle between the observation point and the center of the square source. The above approximations apply explicit restrictions on the size of each square source, *a*, the normal length between the adjacent layers, $z' - z$, relative to the wavelength λ. Notice that only when $z \gg a^2/\lambda$ can the term $(x^2 + y^2)/2(z' - z)$ be neglected. Then the Supplementary Equation 1 can be rewritten as the Fraunhofer diffraction formula [3]:

$$P(\mathbf{r}') = \frac{\mathrm{j}k e^{-\mathrm{j}kr_0}}{2\pi r_0} t^l e^{j\varphi^l} \cos\alpha \iint_{Sq} e^{\mathrm{j}k\left(\frac{xx'+yy'}{z'-z}\right)} \mathrm{d}x\mathrm{d}y, \quad (2)$$

where $r_0 = z' - z + \frac{x'^2+y'^2}{2(z'-z)}$ is the approximation of the length between the observation point and the square source's center. We can easily get the field from Supplementary Equation 2 integral as

$$P(\mathbf{r}') = 4a^2 t^l e^{j\varphi^l} \cos\alpha \frac{\mathrm{j}ke^{-\mathrm{j}kr_0}}{2\pi r_0} \frac{\sin[kax'/(z'-z)]}{kax'/(z'-z)} \frac{\sin[kay'/(z'-z)]}{kay'/(z'-z)}. \quad (3)$$

For a deep-learning neural network, the full connection physically requires each meta-neuron in a specific layer effectively connects to all the meta-neurons in the neighboring layer and can be mathematically described by using an ideal monopole source to replace the square meta-neuron in the following training process. Therefore, the directivity factor $\frac{\sin[kax'/(z'-z)]}{kax'/(z'-z)} \frac{\sin[kay'/(z'-z)]}{kay'/(z'-z)}$ in Supplementary Equation 3 would be trivial only when the size of each square source, a, is much smaller than wavelength. Besides, the axial distance between two adjacent layers and the compactness of the whole system are essentially restricted by the relationship $z \gg a^2/\lambda$. From the analytical analysis above it can be clearly seen that the unique capabilities of metamaterials to provide abrupt phase discontinuity within deep-subwavelength scale are critical for passive neural-networks with small physical scale and objects containing subwavelength details. This represents the fundamental distinction between the mechanisms underlying our meta-neural-network and conventional diffractive neural-network producing large phase variation over distance comparable to wavelength (viz., $a \sim \lambda$). From the comparison between the radiation patterns simulated for two square sources with different size shown in Supplementary Figure 1, we can see at the length of 8λ, the deep-subwavelength square meta-neurons enables the good equivalence between the mathematical interpretation and the meta-neuron network as a result of well-defined monopole source approximation (more details of which are shown in the Supplementary Note 2).

These quantitative results unambiguously suggest the potential of meta-neural-network to recognize objects with device orders of magnitudes more compact than diffractive component neural networks, as will be demonstrated numerically and experimentally in the manuscript (more comparisons of which are shown in the Supplementary Note 4).

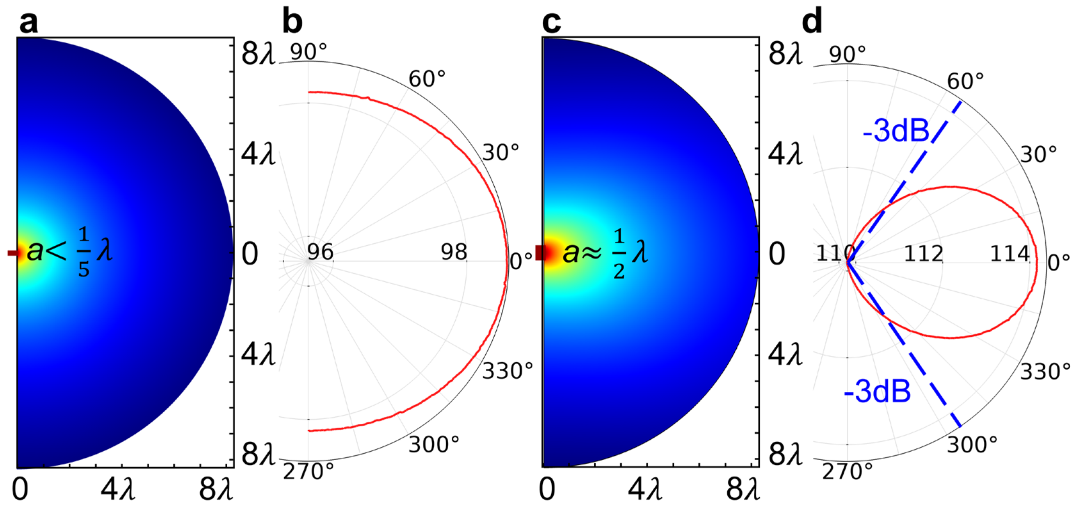

Supplementary Figure 1 | The radiation pattern of a square source in free 3D space. (**a**, **b**) The sound amplitude distribution and polar directivity graph for a square source of size $a < \frac{1}{5}\lambda$; (**c**, **d**) The sound amplitude distribution and polar directivity graph for a square source of size $a \approx \frac{1}{2}\lambda$.

Supplementary Note 2. Forward propagation model and softmax-cross-entropy loss

Due to the deep-subwavelength size of each meta-neuron, it is reasonable to approximately regard it as an ideal point source as demonstrated in Supplementary Note 1. Then the wave propagation function between an arbitrary spatial location and a specific meta-neuron can be expressed as

$$g\left(\mathbf{r}, \mathbf{r}_{m,n}^{l}\right) = \frac{\exp\left(-\mathrm{j}k_0\left|\mathbf{r}-\mathbf{r}_{m,n}^{l}\right|\right)}{4\pi\left|\mathbf{r}-\mathbf{r}_{m,n}^{l}\right|}, \tag{4}$$

where $\mathbf{r}_{m,n}^{l} = (x_m, y_n, z_l)$ refers to the position of this meta-neuron located at m-th column and n-th row on the l-th layer (m=1, 2,…, M, n=1, 2,…, N and l=1, 2, …, L with the total row (column) number M (N) and total layer number L being chosen as 28 (28) and 2 here respectively), with z_l being the position of the l-th layer in the z-axis, $k_0 = \omega/c$ is the wave number, c is the airborne sound velocity and j=$\sqrt{-1}$. Hence, as shown in Supplementary Figure 2, the input wave of the meta-neuron located at the r-th

column and s-th row of next layer (viz. the (l+1)-th layer) can be expressed as

$$p_{r,s}^{l+1} = \sum_{m=1}^{\mathrm{M}} \sum_{n=1}^{\mathrm{N}} g\left(\mathbf{r}_{r,s}^{l+1}, \mathbf{r}_{m,n}^{l}\right) p_{m,n}^{l} W_{m,n}^{l}, \quad (5)$$

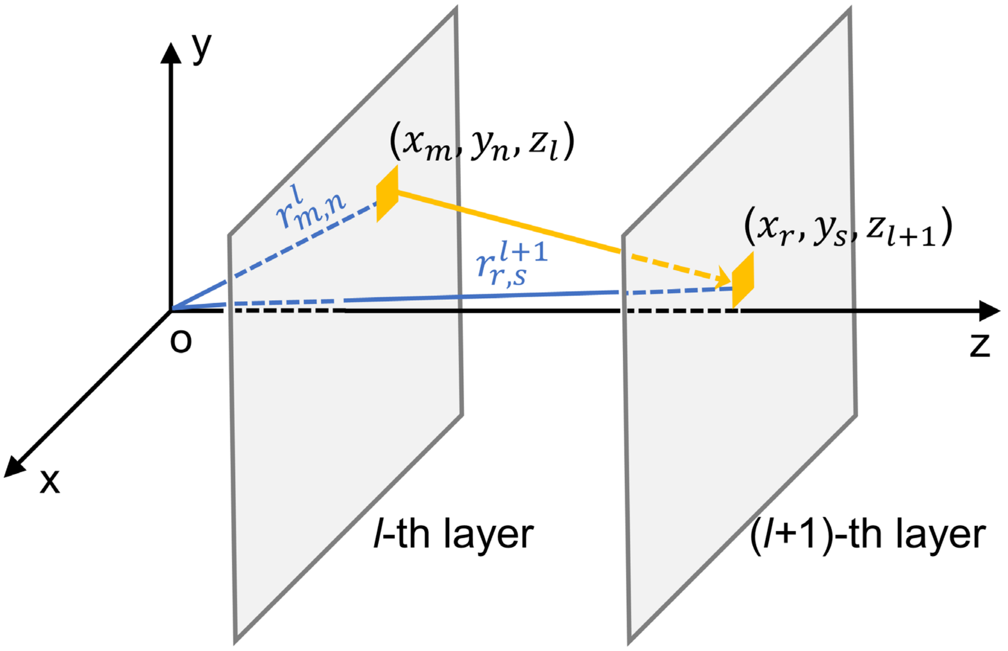

Supplementary Figure 2 | 3D schematic of the forward propagation model in the *l*-th and (*l*+1)-th layers of meta-neurons.

where $p_{r,s}^{l+1}$ is the pressure of the input wave impinging on the meta-neuron located at (x_r, y_s, z_{l+1}), $g\left(r_{r,s}^{l+1}, r_{m,n}^{l}\right)$ is the wave propagation function between these two meta-neurons locating at (x_m, y_n, z_l) and (x_r, y_s, z_{l+1}) respectively, $W_{m,n}^{l}$ is the amplitude and phase modulation of the meta-neuron located at (x_m, y_n, z_l) defined as $W_{m,n}^{l} = t_{m,n}^{l} \exp\left(\varphi_{m,n}^{l}\right)$ with $t_{m,n}^{l}$ and $\varphi_{m,n}^{l}$ being the amplitude and phase modulation respectively. To build the analog between our meta-neural-network with $M \times N$ meta-neurons on each of the L layers and a classic neural-network containing $M \times N \times L$ neurons, we rewrite Supplementary Equation 5 into a more concise form, as follows

$$p_{i}^{l+1} = \sum_{k=1}^{\mathrm{M \times N}} g_{i,k}^{l}\, p_{k}^{l} W_{k}^{l}, \quad (6)$$

where $g_{i,k}^{l} = g\left(r_{r,s}^{l+1}, r_{m,n}^{l}\right), i = M \times (s-1) + r$ and $k = M \times (n-1) + m$. And the incident wave on the first layer of meta-neuron is determined by the input signal and the wave propagation function between the object to be recognized and this layer,

as follows

$$p_i^1 = \sum_{k=1}^{\mathrm{M\times N}} g_{i,k}^0 \, p_k^{\mathrm{I}}.$$

Apparently, Supplementary Equation 6 can be transformed as the operation among vectors

$$\begin{cases} \mathbf{P}^{l+1} = \mathbf{G}^l \cdot (\mathbf{P}^l \circ \mathbf{W}^l), l = 1,2,3,\ldots,\mathrm{L} \\ \mathbf{P}^1 = \mathbf{G}^0 \cdot \mathbf{P}^{\mathrm{I}} \end{cases} \tag{7}$$

where $\mathbf{P}^l = (p_1^l, \ldots\ldots, p_{\mathrm{M\times N}}^l)^{\mathrm{T}}$, $\mathbf{W}^l = (W_1^l, \ldots\ldots, W_{\mathrm{M\times N}}^l)^{\mathrm{T}}$, $\mathbf{P}^l \circ \mathbf{W}^l = (p_1^l W_1^l, \ldots, p_{\mathrm{M\times N}}^l W_{\mathrm{M\times N}}^l)^{\mathrm{T}}$, and

$$\mathbf{G}^l = \begin{pmatrix} g_{1,1}^l & \cdots & g_{1,\mathrm{M\times N}}^l \\ \vdots & \ddots & \vdots \\ g_{\mathrm{M\times N},1}^l & \cdots & g_{\mathrm{M\times N,M\times N}}^l \end{pmatrix}.$$

Hence the acoustic intensity distribution I^{d} on the detection plane can be written as

$$\mathbf{I}^{\mathrm{d}} = (\mathbf{P}^{\mathrm{L}+1})^* \circ \mathbf{P}^{\mathrm{L}+1},$$

where $\mathbf{P}^{\mathrm{L}+1}$ can be derived from Supplementary Equation 7 and $(\mathbf{P}^{\mathrm{L}+1})^*$ is the complex conjugation of $\mathbf{P}^{\mathrm{L}+1}$. By summing up the acoustic intensity in the ten detection regions located on the detection plane, as shown in Fig. 1(**a**) with each covering an area of 14 × 14 cm, $\mathbf{I}^{\mathrm{d}}$ can be transformed into a vector as $(I_0, I_{1,} \ldots, I_9)^{\mathrm{T}}$ in which the elements are the intensity values summed. In the training process, an extra softmax layer is added to transfer the output intensity distribution into the probability distribution of digits[4], which is defined as

$$prob_q = \frac{\exp(I_q)}{\sum_{q=0}^{9} \exp(I_q)}.$$

In general, the mean squared error is often used for function approximation (viz., regression) problems because of its convenience in mathematical analysis, while the cross-entropy error function is commonly more suitable for our interested classification problems when outputs are interpreted as probabilities[5]. The selection of loss function provides different level of effective overparameterization, which greatly affects the final performance[6]. Problems with MSE has also been analyzed and verified in other paper[7], "we have already seen that least-squares solutions lack robustness to outliers, and this applies equally to the classification application." Only when the loss function is appropriately chosen for accurately specifying one's goal of search can satisfactory

results be achieved.

For a quantitative evaluation of the difference between predicted probabilities and ground-truth probabilities, we introduce the cross-entropy loss and try to minimize the difference between probabilities predicted by our meta-neural-network and ground-truth probability via adjustment of the phase modulation conducted by meta-neurons during the training process. The cross-entropy loss function[8] is defined as

$$\text{loss} = -\sum_{q=0}^{9} g_q \log{(prob_q)}$$

g_q is the q-th element at the label corresponding to this digit. It is apparent that by introducing the softmax layer to optimize the training process, we ensure that the criterion of classification is still the region with the maximum acoustic intensity.

In order to verify the correctness of above conclusions in the specific object-recognizing tasks of our interest, here we take an example to explain the reason why MSE loss treating all classes equally is less suitable for classification. In the handwritten digit recognition, when the input digit is '0' (the corresponding label is (1,0,0,0,0,0,0,0,0,0)), and assuming there are two kinds of outputs, one is (1,0.5,0.5,0.5,0.5,0.5,0.5,0.5,0.5,0.5) which means the input digit '0' is correctly recognized, and the other is (0.1,1,0.1,0.1,0.1,0.1,0.1,0.1,0.1,0.1) which means the network incorrectly recognizes the input digit '0' as digit '1'. However, the MSE loss value of the correctly classified output is 2.25, much higher than 1.89 of the incorrect one. From the viewpoint of MSE loss function, the lower loss is the better choice, but unfortunately in reality, it fails to complete the classification task. Intuitively, the MSE loss focuses on the incorrect region in the detection plane too much, and does not punish the misclassified enough, bringing about the higher loss of the correct classified output. In contrast, the cross-entropy loss with an extra softmax layer (also called as 'softmax-cross-entropy loss', SCE loss for abbreviation) is more tolerant of the output in incorrect regions and focuses on the correct region. Taking the above as an example, the SCE loss of the correct classified output is 1.8654 while the incorrect one is 2.4388. The SCE loss chooses the output with lower loss and correctly recognizes the input digit in the meantime. Thus, comparing with the MSE loss, the SCE loss focuses more

on the correct class and tolerant the non-zero intensity in other regions. The authors[6] also prove that the softmax-cross-entropy (SCE) loss performs better than mean square error (MSE) loss due to the fact that "QL (quadratic loss, the definition is the same with MSE) focuses on fitting all classes, whereas cross-entropy (CE) focuses on only the correct class (associated with the label)".

Supplementary Note 3. Role of the wave propagation function

From the above derivation, the forward propagation function between two neighboring layers can be written as

$$\mathbf{P}^{l+1} = \mathbf{G}^{l} \cdot (\mathbf{P}^{l} \circ \mathbf{W}^{l}), \tag{8}$$

where $\mathbf{P}^{l}$ denotes the input wave of the l-th metasurface, $\mathbf{G}^{l}$ is the wave propagation matrix, $\mathbf{W}^{l} = \mathbf{t}^{l}\exp(\boldsymbol{\varphi}^{l})$ is the amplitude and phase modulation introduced by the meta-neurons at l-th metasurface with $\mathbf{t}^{l}$ and $\boldsymbol{\varphi}^{l}$ being the amplitude and phase modulations respectively, and '∘' denotes the element-wise multiplication. While the conventional neural network can be written as

$$\mathbf{Y}^{l+1} = f(\mathbf{w}^{l} \cdot \mathbf{Y}^{l} + \mathbf{B}^{l}), \tag{9}$$

where f is the nonlinear active function, $\mathbf{w}^{l}$ is the weight and $\mathbf{B}^{l}$ is bias.

Comparison of Supplementary Equations 8 and 9 clearly reveals the equivalence between our proposed meta-network and a conventional neural network. To be specific, the learnable parameters in our meta-neural-network are the phase modulation provided by the meta-neurons, and the wave propagation function between two neighboring layers of meta-neurons prevents the multi-layered meta-neural-network from degenerating into a monolayer meta-neural-network in physical systems. As a result of such equivalence, one can strictly prove that in our meta-neural-network, each meta-neuron connects to all the meta-neurons on the neighboring layer.

In order to verify the role of wave propagation matrix $\mathbf{G}$, we use a bilayer meta-neural-network as an example, and prove that a monolayer meta-neural-network could not replace a bilayer one with nonzero physical distance between these two layers. Similar to Supplementary Equation 8, the pressure distribution on detection plane is

$$\mathbf{P}^{d} = \mathbf{G}^{2} \cdot (\mathbf{P}^{2} \circ \mathbf{W}^{2}) = \mathbf{G}^{2} \cdot ((\mathbf{G}^{1} \cdot (\mathbf{P}^{1} \circ \mathbf{W}^{1})) \circ \mathbf{W}^{2}). \tag{10}$$

Therefore, in order to find a monolayer meta-neural-network for replacing this two-layer meta-neural-network, we need to seek a specific vector $\mathbf{W}$ that makes the following relationship stands

$$\mathbf{P}^{d1} = \mathbf{G}^2 \cdot (\mathbf{P}^1 \circ \mathbf{W}) = \mathbf{P}^d. \tag{11}$$

In other words, physically we have to build a single hidden layer with a learnable vector W that perfectly mimics the wave field produced by these two layers and the wave propagation function between them.

To simplify the derivation, we rewrite Supplementary Equation 8 as

$$\mathbf{P}^{l+1} = \mathbf{G}^l \cdot (\mathbf{P}^l \circ \mathbf{W}^l) = (\mathbf{G}^l \circ f((\mathbf{W}^l)^T)) \cdot \mathbf{P}^l, \tag{12}$$

where $(\mathbf{W}^l)^T$ means the transpose of the vector, $f(x)$ is a function that expands a vector into a matrix. If x is a row vector $(x_1, x_2, \dots\dots, x_n)$, then $f(x)$ is

$$\begin{pmatrix} x_1 & x_2 & \cdots & x_{n-1} & x_n \\ x_1 & x_2 & \cdots & x_{n-1} & x_n \\ \vdots & \vdots & \ddots & \vdots & \vdots \\ x_1 & x_2 & \cdots & x_{n-1} & x_n \\ x_1 & x_2 & \cdots & x_{n-1} & x_n \end{pmatrix}.$$

Thus Supplementary Equation 12 can be rewritten as

$$\mathbf{P}^{l+1} = \mathbf{H}^l \cdot \mathbf{P}^l,$$

where $\mathbf{H}^l = \mathbf{G}^l \circ f((\mathbf{W}^l)^T)$.

Similarly, Supplementary Equation 10 can be rewritten as

$$\mathbf{P}^d = \mathbf{H}^2 \cdot \mathbf{P}^2 = \mathbf{H}^2 \cdot \mathbf{H}^1 \cdot \mathbf{P}^1 = \mathbf{H} \cdot \mathbf{P}^1,$$

where $\mathbf{H}^2 = \mathbf{G}^2 \circ f((\mathbf{W}^2)^T)$, $\mathbf{H}^1 = \mathbf{G}^1 \circ f((\mathbf{W}^1)^T)$, $\mathbf{H} = \mathbf{H}^2 \cdot \mathbf{H}^1$.

And Supplementary Equation 11 can be rewritten as

$$\mathbf{P}^{d1} = \mathbf{G}^2 \cdot (\mathbf{P}^1 \circ \mathbf{W}) = \left(\mathbf{G}^2 \circ f(\mathbf{W}^T)\right) \cdot \mathbf{P}^1 = \mathbf{P}^d = \mathbf{H} \cdot \mathbf{P}^1. \tag{13}$$

Assuming there exists a specific $\mathbf{W}$ such that

$$\mathbf{G}^2 \circ f(\boldsymbol{W}^T) = \mathbf{H}, \tag{14}$$

by expanding Supplementary Equation 14 as

$$\mathbf{G}^2 \circ f(\mathbf{W}^T) = \begin{pmatrix} G_{11}^2 W_1 & G_{12}^2 W_2 & \cdots & G_{1(n-1)}^2 W_{n-1} & G_{1n}^2 W_n \\ G_{21}^2 W_1 & G_{22}^2 W_2 & \cdots & G_{2(n-1)}^2 W_{n-1} & G_{2n}^2 W_n \\ \vdots & \vdots & \ddots & \vdots & \vdots \\ G_{(m-1)1}^2 W_1 & G_{(m-1)2}^2 W_2 & \cdots & G_{(m-1)(n-1)}^2 W_{n-1} & G_{(m-1)n}^2 W_n \\ G_{m1}^2 W_1 & G_{m2}^2 W_2 & \cdots & G_{m(n-1)}^2 W_{n-1} & G_{mn}^2 W_n \end{pmatrix}$$

$$= \begin{pmatrix} H_{11} & H_{12} & \cdots & H_{1(n-1)} & H_{1n} \\ H_{21} & H_{22} & \cdots & H_{2(n-1)} & H_{2n} \\ \vdots & \vdots & \ddots & \vdots & \vdots \\ H_{(m-1)1} & H_{(m-1)2} & \cdots & H_{(m-1)(n-1)} & H_{(m-1)n} \\ H_{m1} & H_{m2} & \cdots & H_{m(n-1)} & H_{mn} \end{pmatrix} = \mathbf{H},$$

one can arrive at a relationship W_1 needs to satisfy

$$W_1 = \frac{H_{11}}{G_{11}^2} = \frac{H_{21}}{G_{21}^2} = \cdots = \frac{H_{(m-1)1}}{G_{(m-1)1}^2} = \frac{H_{m1}}{G_{m1}^2}. \tag{15}$$

Clearly there is no value satisfying all the equivalence of Supplementary Equation 15 in most cases unless the layer distance between two hidden layers is 0. When the axial distance between the two layer is zero, $\mathbf{G}^1$ is an identity matrix and Supplementary Equation 13 becomes

$$\mathbf{H} = \mathbf{H}^2 \cdot \mathbf{H}^1 = \mathbf{G}^2 \circ f((\mathbf{W}^2 \circ \mathbf{W}^1)^T) = \mathbf{G}^2 \circ f((\boldsymbol{W})^T).$$

As a result, only when the distance between two neighboring layers is zero can we find the matrix $\mathbf{W} = \mathbf{W}^2 \circ \mathbf{W}^1$ required by the degeneracy of the bilayer meta-neural-network into a monolayer one (apparently, in such a case the two layers in this physical model literally merges into a single layer)

As a consequence, $\mathbf{G}$ prevents the multi-layered meta-neural-network from degenerating into a monolayer meta-neural-network in physical system and thus plays a significant role in the meta-neural-network.

Moreover, the axis distance which determined the wave propagation function is not necessary to optimize during the training process since the axis distance will not appreciably improve the classification performance (see Supplementary Figure 3), as evidence by the results showing nearly stable performance for axis distance varying within a large range above this lower-limit, which significantly simplifies the design of

our meta-neural-network. Thus, the wave propagation function which prevents the multi-layered meta-neural-network from degenerating into a monolayer meta-neural-network in physical system and forms the connection between adjacent layers is a hyperparameter rather than learnable parameter.

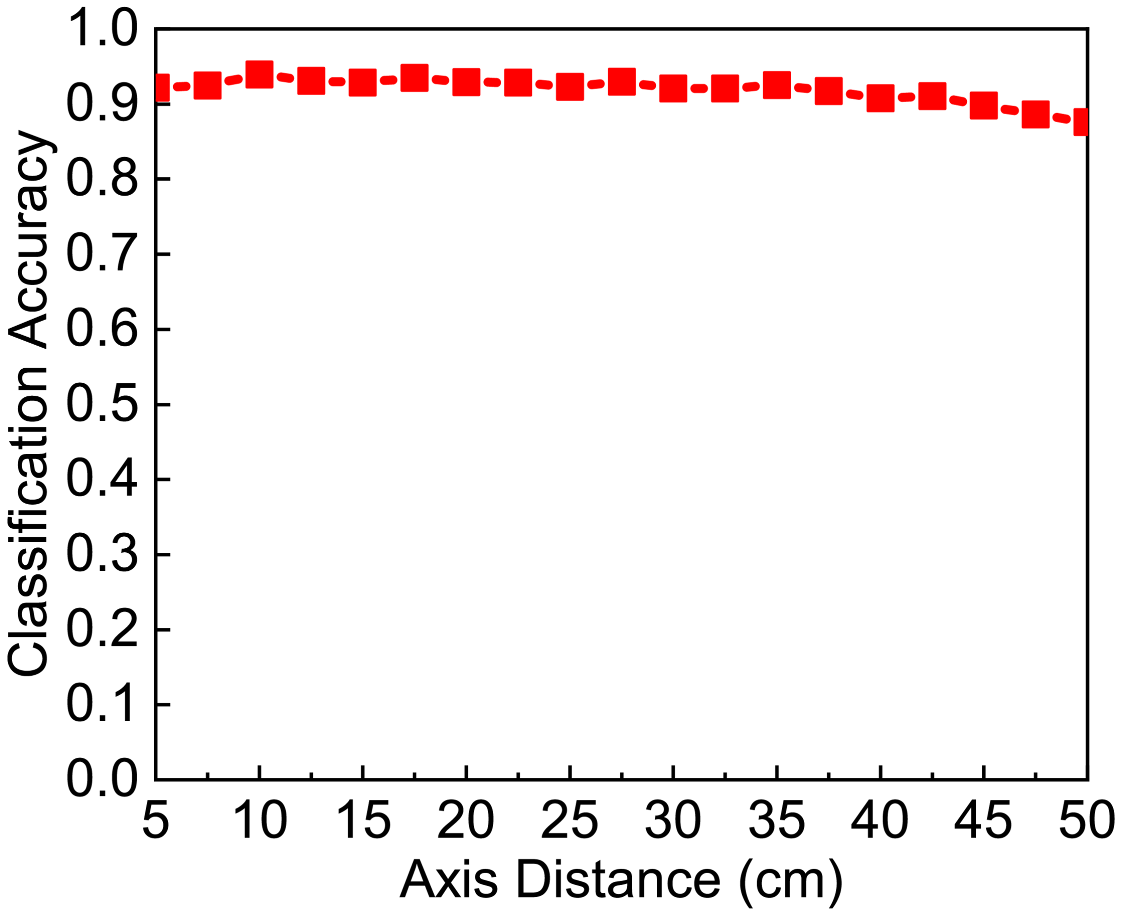

Supplementary Figure 3 | The classification accuracy of a bilayer meta-neural-network as a function of axis distance.

In this work, we do not introduce nonlinear response to mimic the nonlinear activation function in conventional neural network, however, by employing programmable active metamaterials[9], or tunable acoustic switch[10], it is possible to realize nonlinear activation function in our physical model. The acoustic devices above serving as acoustic switches can be manipulated by secondary sound or AC voltage. By combining the meta-neurons with such acoustic switchable devices controlled by sound or other external energy sources, both the learnable parameters and nonlinear activation function can be simultaneously introduced into the system. This would be the goal of our future work.

Supplementary Note 4. The comparison between meta-neural-network and diffractive neural network in compact systems

To demonstrate the key importance of deep-subwavelength nature of meta-neurons, here we train a series of meta-neural-network for which the thickness of each meta-neuron layer is unchanged but the width of a single building block becomes one half-wavelength (6cm) and the layer distance is chosen such that the monopole approximation stands. Accordingly, the meta-neuron number in one layer becomes 10×10 (100 in total). The training result is shown in Fig. 2(**b**) which clearly reveals that despite the subwavelength size of meta-neuron, this meta-neural-network is far outperformed by meta-neural-network composed of meta-neurons downscaled to deep-subwavelength regime. This indicate that deep-subwavelength structure is vital for the miniaturization of devices and its application for small objects.

In addition, we need to stress that the diffractive elements cannot precisely provide the abruptly-changing phase profile under the mathematical framework proposed in the current work for realizing an ideal passive neural network, leading to the fact that the training results of such meta-neural-network of half-wavelength-sized neurons, shown in Fig. 2(**b**), cannot be outperformed by diffractive layers with equal size. Supplementary Figure S4 shows the desired distribution calculated from the mathematical model described in Supplementary Note 1 and the intensity distributions produced by meta-neural-network and diffractive-neural-network, both of which have the neuron size of one half-wavelength. From the results one can clearly observe that under this circumstance, the equivalence between the mathematical interpretation and the meta-neuron-network is well established despite the relatively large unit cell while the diffractive layer is not capable of precisely mimicking a standard neural network and directing the acoustic energy into the desired region as expected.

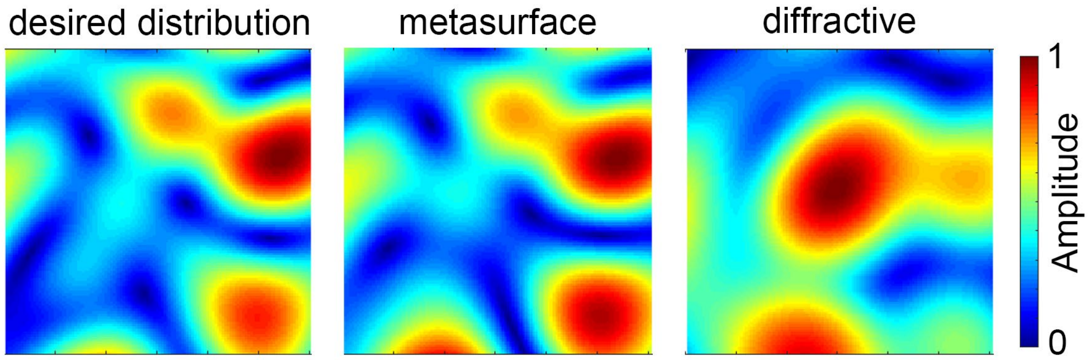

Supplementary Figure. 4 | The comparison among the desired intensity distribution on detection plane and the intensity distributions produced by a meta-neural-network and a passive neural network based on diffractive layers.

We further investigate essential difference between meta-neural-network and passive neural network build on diffractive layers and demonstrate the typical simulated results of comparison of intensity distributions on detection plane produced by these two systems with equal-sized bilayer structure (5×5 wavelength) and same neuron number (28×28) respectively, for particular objects chosen as ten handwritten digits, as shown in Supplementary Figure 5. We can see that the energy going through the meta-neural-network is accurately redistributed into the expected region corresponding to the handwritten digits. In stark contrast, the output pattern produced by diffractive neural network is severely blurred and apparently cannot be recognized accurately as the corresponding digit, due to the fact that diffractive elements cannot provide the required subwavelength-distribution of abrupt phase shift. This clearly indicates the extraordinary capability of our proposed meta-neural-network to work for device and object downsized to scales orders of magnitudes smaller than achievable with diffractive neural-network that cannot ensure production of the arbitrary and discrete phase distribution yielded by the training process which is vital for the equivalence between mathematical neural network model and practical physics system.

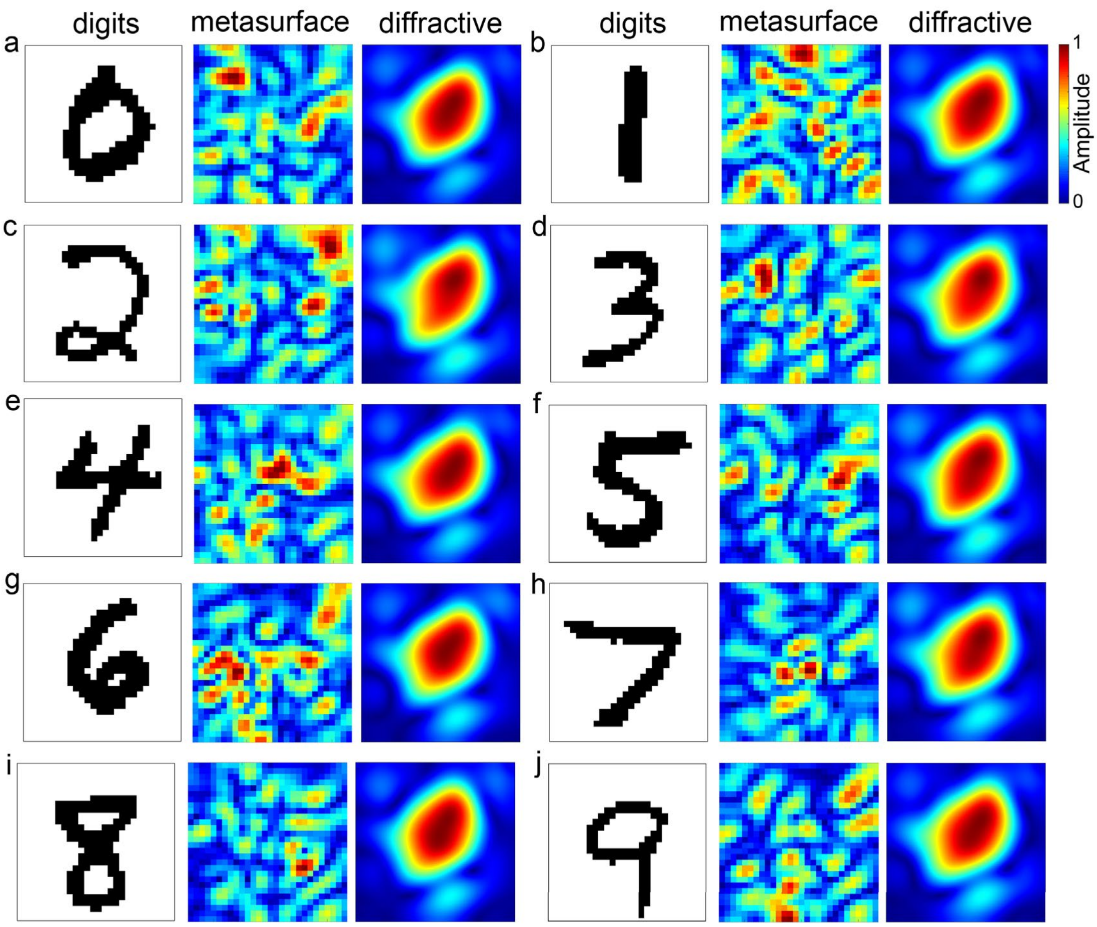

Supplementary Figure 5 | The comparison of the distribution of intensity on detection plane produce by a meta-neural-network and a passive neural network based on diffractive layers. (**a-j**) show the acoustics intensity distributions of ten handwritten digits on the detection plane produced by meta-neural-network and diffractive neural network. The total acoustic intensity has been normalized with respect to the maximal value measured in all the detection regions assigned to the ten digits.

Supplementary Note 5. The design of acoustic meta-neurons

In the current study, we implement the meta-neuron by using a specific kind of acoustic metamaterial unit cell composed of four local resonators and a straight pipe. In this design, the series connection of cavities supports strong local resonance for effectively slowing down the propagation of wave as it passes by, enabling free control of the propagation phase within the full 0-to-2π range. On the other hand, the impedance mismatch caused by these local resonators is maximally compensated by the

introduction of the straight pipe supporting Fabry-Perot resonance, which results in hybrid resonance and ensures full 2π control of phase shift while keeping a near-unity transmission efficiency. This is verified by the numerical results depicted in Supplementary Figure 6(c), which shows that the phase shift can be smoothly tuned within the range from 0 to 2π by adjusting a single structural parameter, h ,which is the height of pipe, with no need of changing the overall size of each subwavelength meta-neuron (2 cm in width and 7 cm in thickness). It is also noticed that such a metamaterial design leaves an average of 50% cross-section open for each layer and keeps the continuity of the background medium, which allows other entities such as light or flow to pass and helps to improve the application of the resulting devices in practical scenarios such as photoacoustic imaging where the opaque acoustic transducers usually block the transmission of light. Based on the parameter dependence of phase shift given by the simulation results shown in Supplementary Figure 6(c), we determined the precise geometric parameter for each meta-neuron and fabricated a meta-neural-network comprising two layers with transversal size of 56×56 cm.

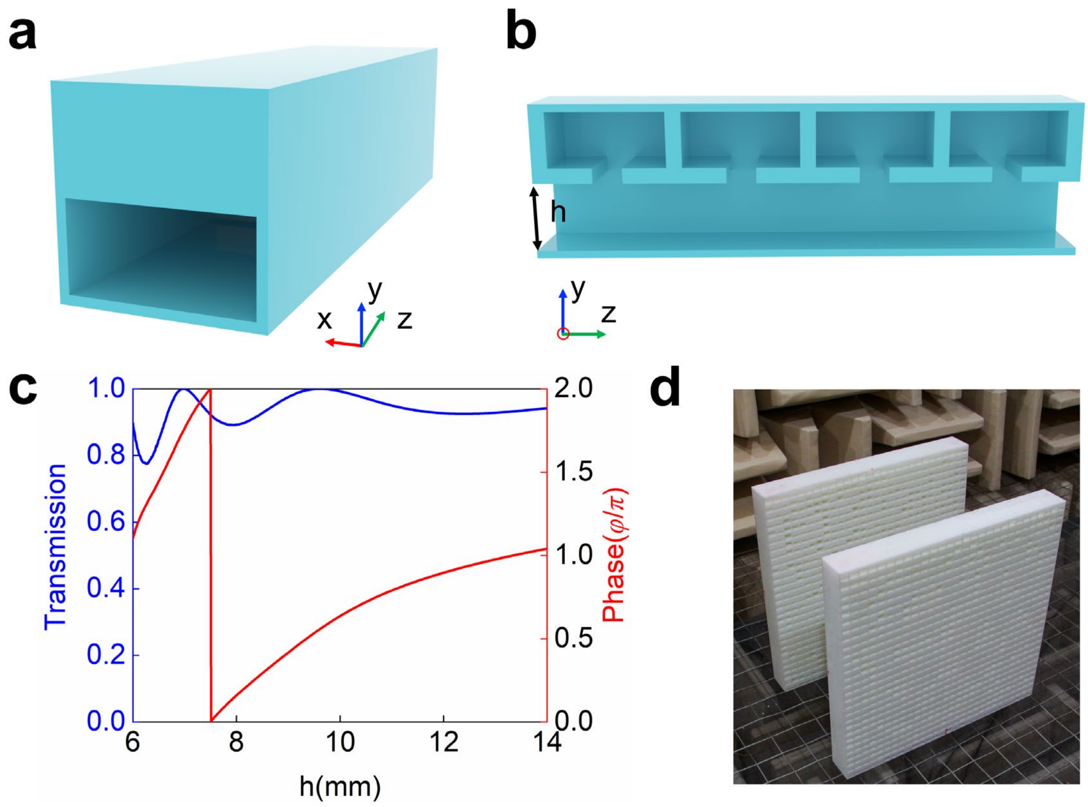

Supplementary Figure 6 | Implementation of meta-neuron as basic building

block of the proposed acoustic meta-neural-network. (**a**) 3D schematic view of an individual meta-neuron which is a metamaterial unit cell with a hybrid structure. (**b**) shows the 2D cross-section view of the meta-neuron illustrated in (A) which is formed by coupling four local resonators and a straight pipe for producing a coupled resonance that compensates the impedance mismatch in the phase modulation. (**c**) The simulated phase discontinuity provided by this unit cell as a function of the height of pipe, h, shows that adjustment of this single parameter ensures full 2π phase control while keeping near-unity transmission efficiency. (**d**) The photo of the meta-neural-network prototype fabricated via 3D printing technique, which are composed two layers of meta-neurons implemented by metamaterial unit cells with subwavelength size in all dimensions.

Supplementary Note 6. The influence of imperfect transmission efficiency of meta-neurons on the classification accuracy

In the design of our proposed meta-neural-network, an ideal meta-neuron is assumed to provide a phase shift within the full range from 0 to 2π, which is chosen as the the learnable parameter in the training process, while keeping unity transmission for the incident wave. In spite of the fact that full manipulation of sound field requires independent control of both amplitude and phase[11], we numerically prove that an acoustic meta-neural-network comprising such phase-only meta-neurons suffices to recognize the digit-shaped objects with high accuracy and, meanwhile, significantly reduce the difficulty of sample design and fabrication. In the experiments, we implement our strategy by designing a hybrid metastructure composed of four side-loaded resonators and a straight pipe. Due to the efficient coupling between the resonances in the cavities and pipe that effectively compensates the impedance mismatch to the surrounding air, such meta-neuron enables full 2π control of propagation phase and near-unity acoustic transmission (>90%) and satisfyingly mimics a meta-neuron with the above assumption. Nevertheless, one can observe in Supplementary Figure 6(c) in Supplementary Note 5 that the transmission of meta-neurons with different phase shift still varies slightly. It is therefore necessary to give a

precise estimation on how the object-recognition performance of the resulting meta-neural-network will be affected by the imperfect transmission of basic building blocks. To this end, we numerically calculate the output of meta-neural-network by taking into consideration the practical transmission efficiency of each unit cell, and depict the typical results in Supplementary Figure 7. As shown by the result, in comparison to the meta-neural-network consisting of ideal meta-neurons, there is no appreciable reduction in the accuracy of object recognition and classification for a practical meta-neural-network, proving that the error caused by neglecting the amplitude variation is trivial. This is also verified by the high accuracy exhibited by the meta-neural-network prototype in the experimental measurements.

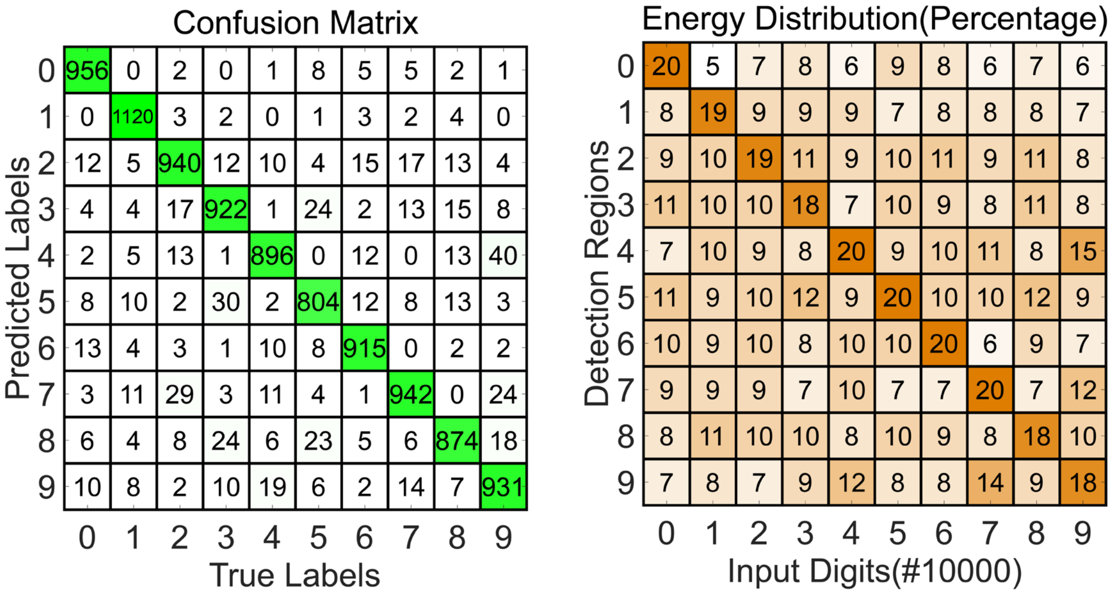

Supplementary Figure 7 | (a) The confusion matrix and (**b**) energy distribution for bilayer meta-neural-networks with the transmission efficiency of practical meta-neurons being taken into account.

Supplementary Note 7. Preparation of the input data for the meta-neural-network

In the current study, we choose to demonstrate the unique functionality of our proposed meta-neural-network via passive and real-time recognition of objects with shape of handwritten digits, and accordingly employ the MNIST (Modified National Institute of Standard and Technology) database[12] that is a large database commonly used for training and testing the recognition of handwritten digits. The MNIST database

contains 55,000 training images, 5,000 validation images and 10,000 testing images. Each row vector in the MNIST database has a length of 784 and can be transformed into a 28×28 matrix, with the value of each matrix element representing the grayscale value of the pixel at the same position of a square 28×28 image. Each image in the MNIST database corresponds to a label indicating which digit this image represents. Each label is a 1×10 vector consisting of one "1" and nine "0", with the location of "1" referring to the digit. For example, the labels are (1 0 0 0 0 0 0 0 0 0) and (0 0 0 0 0 1 0 0 0 0) for digits "0" and "5" respectively. Given that all the MNIST images are grayscale images which are not suitable for the design and fabrication of samples in the subsequent experiments, here we binarize all the training images in the database by rounding up the grayscale value for each pixel as illustrated in Supplementary Figure 8(**c**). Instead of simply inputting each image itself into the artificial neural network for training the learnable parameters as usually done in previous works with purpose of image recognition, we use the simulated scattered wave produced by each object which is a solid plate with shape of the dark region in the corresponding binary image (shown in Supplementary Figure 8(**d**)) as the input data in the computer-aided training process.

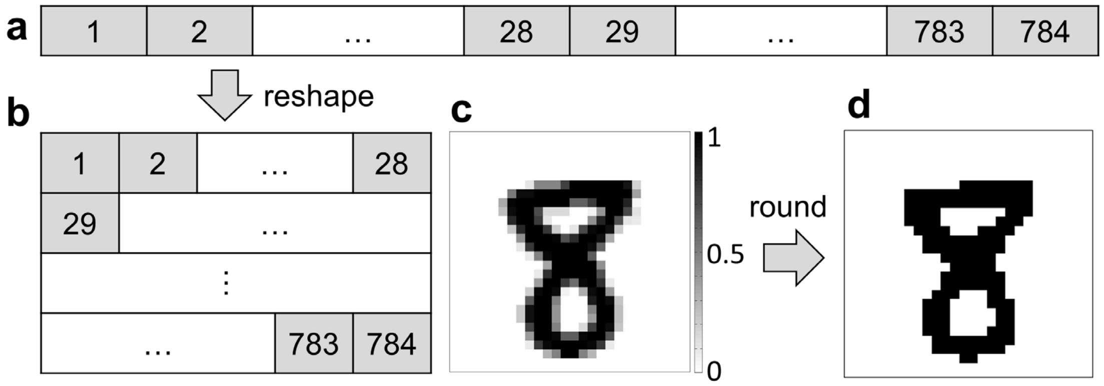

Supplementary Figure 8 | The preparation of an individual digit-shaped object for recognition. (**a**) A row vector in the MNIST database containing 784 elements. (**b**) A 28×28 matrix obtained by shaping the row vector in (a) corresponds to a square 28×28 grayscale image with the grayscale value of each pixel being represented by the value of the matrix element at the same location. (**d**) The binary image formed by rounding up the grayscale value of

each pixel in the image in (**c**), which will be used for the design and fabrication of experimental sample chosen as a thin plate whose shape is the same as the dark region.

Supplementary Note 8. Training details and experimental results of object recognition by acoustic meta-neural-network

Our acoustic meta-neural network was simulated using MATLAB and trained in a desktop with a GeForce RTX 2070 Graphical Processing Unit(GPU), Intel(R) Xeon(R) CPU E5-2620 v3 @ 2.40 GHz and 160 GB of RAM, running Windows 7 operating system(Microsoft). The classification accuracy of testing data keeps increasing and eventually becomes stable within 6 epochs, which took about 3.2 hours to train for our meta-neural-network.

The schematic diagram of the experimental setup is given in Fig. 1(**a**). In the experiment, the input sound was generated by a speaker (Beyma CP380), driven by the waveform generator (RIGOL DG1022). The sensor we used on the detection plane was 1/4-inch free field microphone (BRÜEL & KJÆR Type 4961) and the stand-alone recorder (BRÜEL & KJÆR Type 3160-A-022).

In our proposed mechanism for designing a meta-neural-network, the classification criterion of a specific digit-shaped object is that the total acoustic energy we can gather in the desired detection region assigned to this digit is higher than in the rest regions. Thanks to this design, there is no need to obtain the fine spatial distribution of acoustic intensity within the detection region which usually has to be performed by using a complicated array comprising a large number of sensors or moving the sensor spatially for a point-by-point scanning of the acoustic field. Instead, at the receiving end we conveniently measure the total acoustic intensity in each detection region by using fix number of sensors. By attaching the sensor to the small throat of tapered structure with an exponential profile and a square cross-section which acoustically behaves like a near-reflectionless acoustic energy concentrator, we use a single sensor to realize the energy integration over a specific detection region. In addition, the total sensor number is as few as the number of object classifications (equal to the number of

detection regions and chosen as 10 here) regardless of the resolution or overall size of the meta-neural-network, which is a unique advantage over conventional active-device-based deep learning mechanisms. The typical experimental results of the total acoustic intensity measured on all the detection regions for ten specific objects with shape of handwritten digits from 0 to 9 are depicted in Supplementary Figure 9, which verify the passive, real-time and sensor-scanning-free object-recognizing functionality of the fabricated meta-neural-network that engineers the wavefront of scattered wave by the object and generates the highest total acoustic intensity in the desired detection region.

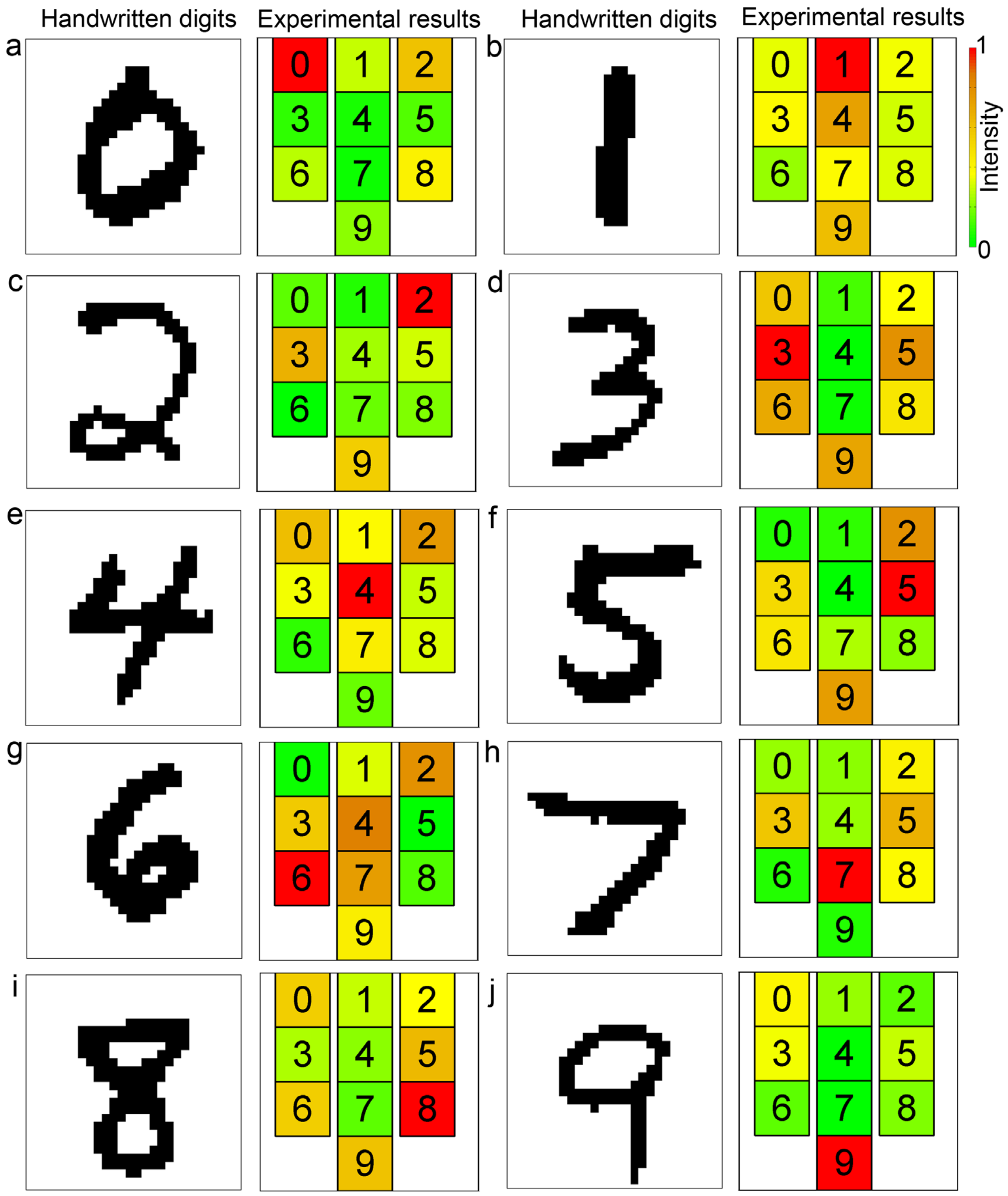

Supplementary Figure 9 | Typical experimental results of accurate object recognition by the fabricated meta-neural-network. (**a-j**) show the total acoustic intensity measured on the detection plane (right column) for ten specific objects (left column) with shape of handwritten digits from 0 to 9, respectively. The total acoustic intensity has been normalized with respect to the maximal value measured in all the detection regions assigned to the ten digits.

Supplementary Note 9. The influence of experimental error in phase shift of meta-neurons on the classification accuracy

In the measurements, we have tested 20 objects with shape of handwritten digit (viz. 2 for each digit), and the experimental results show that the meta-neural-network prototype recognized all the digits accurately except that the two digits "4" are mistakenly recognized as "3". We believe that such incorrect recognition primarily stems from the experimental errors especially the imperfect fabrication of metamaterial sample. In the experiment, we fabricate the meta-neural-network prototype via 3D printing technique, by using a machine with precision of 0.1 mm. Considering the actual size of an individual meta-neuron used in the experiment, such a fabrication error will lead to a uncertainty in the phase shift within the range of $\pm\pi/18$, which is sufficiently large to cause an appreciable move of the output focal region from detection region corresponding to "4" to region "3" when the meta-neural-network interacts with the scattered wave produced by the object. Due to the difficulty of precisely obtaining the actual phase profiles provided by the practical sample of meta-neural-network containing a large number of unit cells (exceeding 1500), we investigate the potential reason responsible for the misclassification of the digit "4" only via numerical simulations. As a result, we numerically prove that when an extra phase shift within $\pm\pi/18$ is introduced for each meta-neuron, which mimics the experimental error in practical situation where the phase profile of the whole meta-neuron layer cannot be controlled perfectly, the resulting meta-neural-network tends to recognized the vast majority of digits "4" as "3" mistakenly while keeping the classification accuracy of all

the rest digits unaffected. And we have successfully found a large number of specific examples of such phase profiles. For verifying the validity of this assumption, we have further fabricated 5 more samples of digit “4” that are numerically proven recognizable and experimentally characterized their classification accuracy. The experimental results show that our practical meta-neural-network prototype only accurately recognized 1 of all the 7 digit “4” samples, and all the misclassified “4” are recognized as “3” as expected. We therefore conclude that this problem will not affect the effectiveness of our mechanism and can be easily solved by increasing the fabrication precision of our meta-neural-network prototype.

Supplementary Note 10. The recognition of multiplexed orbital angular momentum beams

With infinite dimensionality of the Hilbert space, orbital angular momentum (OAM) offers the possibility to dramatically improve the capacity of waves as information carriers and particularly crucial for acoustic waves that dominate underwater communications [13,14]. However, the de-multiplexing of such spatially-multiplexed information carried by many twisted beams with different topological charges (TCs) essentially needs accurate recognition of spatial pattern far more complicated than the above digit-shaped objects can produce. In contrast to the active de-multiplexing mechanisms relying on elaborated sensor array and time-consuming postprocessing [13], purely-passive paradigms offer a simple and low-cost solution for real-time de-multiplexing without sensor scanning or postprocessing (such as by using Dammann gratings, Q-plates, or metasurfaces[14-16]), but still suffers from uncontrollable spatial locations of output beams and, in particular, misalignment between transmitter and receiver that will lead to severe inter-channel crosstalk. Here we demonstrate that our meta-neural-network offers a new mechanism capable of going beyond these fundamental barriers and recognizing TCs in real-time from non-aligned OAM beams.

In the current study, we use a four-layer meta-neural-network ($101 \times 101 \times 4$, 40804 meta-neurons in total) to recognize multiplexed acoustic vortex beams with 8 different TCs ($\pm 1, \pm 2, \pm 3, , \pm 4$). The amplitude and phase distribution at the generating

plane of m-th OAM beams is written as $(B_m(x,y), \phi_m(x,y))$, where $B_m(x,y)$ is the normalized Bessel function and $\phi_m(x,y)$ is the phase shift at x-y plane. Supplementary Figure 10 shows the amplitude and phase distribution of 8 states of OAM beams after propagating a distance of 5 meters. The misalignment between the centres of OAM beam and meta-neural-network is set to be independent along two orthogonal directions (r,θ) (viz., along the radial direction and vertical to the radial direction). The ranges of r and θ are chosen as [0,6λ] and [0,2π) respectively, which represents a significant misalignment considering the side length of metasurface is nearly 18λ.

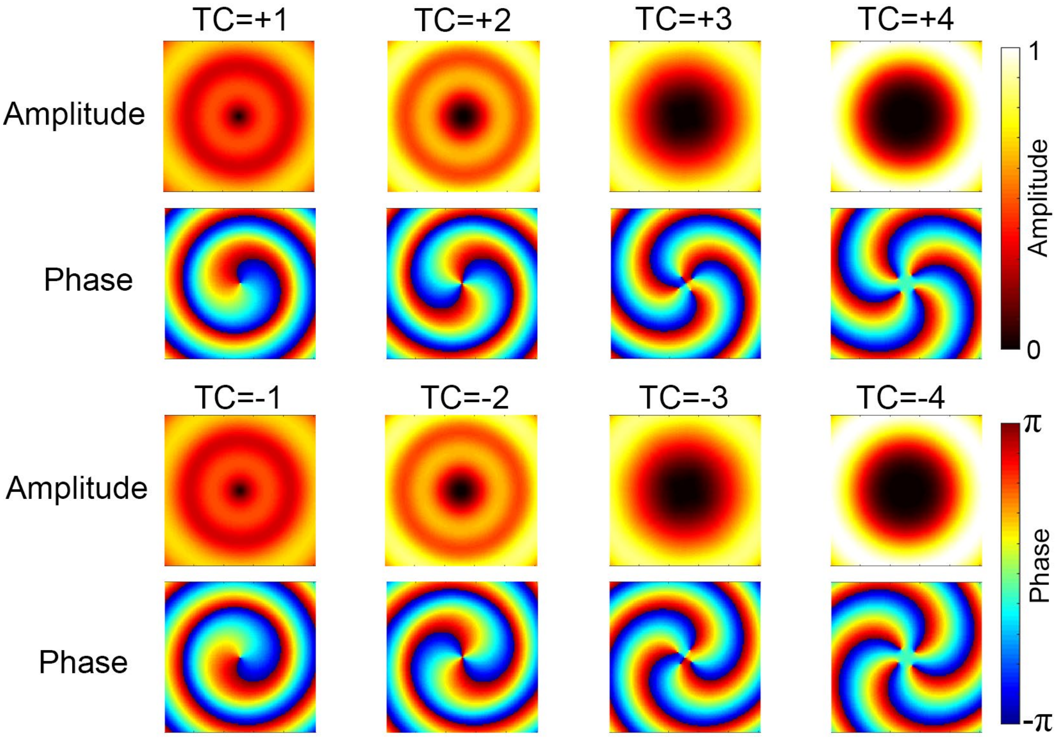

Supplementary Figure 10 | The amplitude and phase distributions of 6 different OAM beams with topological charges of ($\pm1, \pm2, \pm3, \pm4$).

Firstly, we calculate the pressure field of multiplexed OAM beams after they propagate 500 cm and 700 cm in free space respectively, and set these pressure fields as the input of first layer as shown in the Fig. 4(a). Secondly, the misalignment between the centres of OAM beams and meta-neural-network is set to be independent along two orthogonal directions (r,θ) (viz., along and vertical to the radial direction). Supplementary Figure 11(a) demonstrates the maximal misalignment range allowed by

our current design of meta-neutral-network for recognizing three multiplexed OAM beams composed of TCs=$+3, \pm4$ (marked by the green circle), and the pressure distribution of a typical OAM beam with misalignment chosen as $(r = 6\lambda, \theta = \pi)$. Supplementary Figure 11(b) illustrates the zoom-in view of the pressure distribution in the blue square region in Supplementary Figure 11(a) as the input of first layer, which is obviously identical as Fig.4(a) in the manuscript. Last but not least, we use pressure distribution in complex values as the raw data in training. To make sure that the dataset covers sufficiently large misalignments, the ranges of r and θ are chosen as $[0, 6\lambda]$ and $[0, 2\pi)$ respectively, which can be regarded significant enough given that the side length of metasurface is about 18λ. Following this procedure, we generate training data with 80000 multiplexed OAM beams and 10000 testing ones.

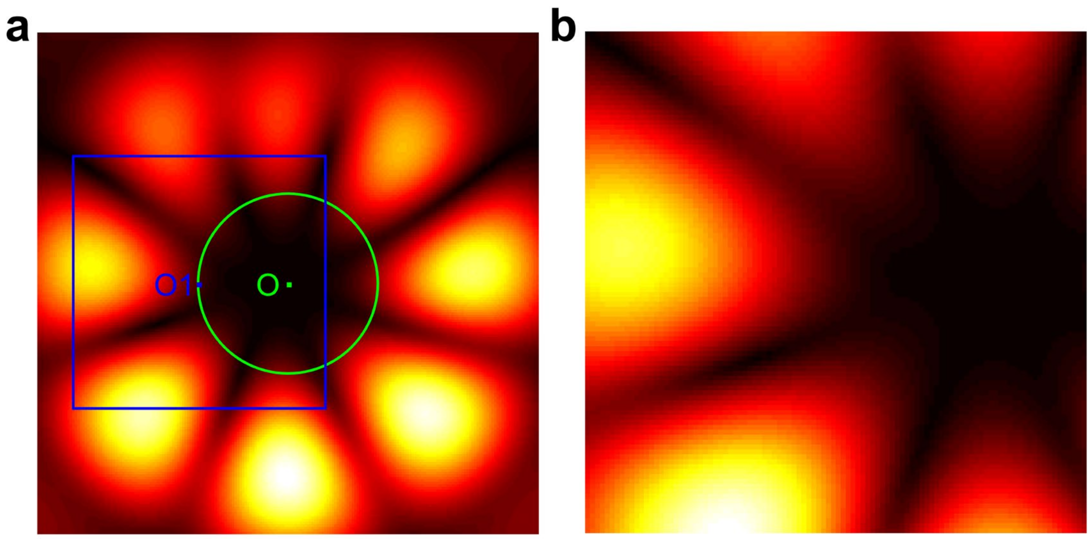

Supplementary Figure 11 | The misalignment between the centre of OAM beams and the centre of metasurface.

The training process of the recognition of multiplexed OAM beam is similar to the recognition of handwritten digits mentioned above, except the criterion of recognition. In this task, the detection plane is divided into eight regions representing eight modes respectively. Notice that the assignment of detection region location is entirely freestyle and independent of TC. Each region has two areas marked by 'Y' and 'N' as shown in Fig. 4(**a**). The existence of a specific OAM mode is judged by comparing the magnitude of total sound energy within these two areas in the corresponding region. If sound

energy in area ‘Y’ is higher, the meta-neural network predicts that this mode is existing in the input wave, and vice versa.

Supplementary References

1 Fu, Y. *et al.* Reversal of transmission and reflection based on acoustic metagratings with integer parity design. *Nature Communications* **10**, 2326 (2019).

2 Born, M. A. X. & Wolf, E. Principles of optics: electromagnetic theory of propagation, interference and diffraction of light[M]. *Elsevier* (2013).

3 Ishimaru A. Electromagnetic wave propagation, radiation, and scattering: from fundamentals to applications[M]. *John Wiley & Sons* (2017).

4 Goodfellow I, Bengio Y, Courville A, et al. Deep learning[M]. *Cambridge: MIT press* (2016).

5 Reed R, MarksII R J. Neural smithing: supervised learning in feedforward artificial neural networks[M]. *Mit Press* (1999).

6 Demirkaya A, Chen J, Oymak S. Exploring the Role of Loss Functions in Multiclass Classification[C]. 2020 54th Annual Conference on Information Sciences and Systems (CISS). 1-5 (2020).

7 Bishop C M. Pattern recognition and machine learning[M]. *springer* (2006).

8 Kroese D P, Rubinstein R Y, Cohen I, et al. Cross-entropy method. *Encyclopedia of Operations Research and Management Science*, 326-333 (2013).

9 Xiao, S., Ma, G., Li, Y., Yang, Z. & Sheng, P. Active control of membrane-type acoustic metamaterial by electric field. *Applied Physics Letters* **106**, 091904 (2015).

10 Vanhille, C. & Campos-Pozuelo, C. An acoustic switch. *Ultrason Sonochem* **21**, 50-52 (2014).

11 Zhu, Y. *et al.* Fine manipulation of sound via lossy metamaterials with independent and arbitrary reflection amplitude and phase. *Nature communications* **9**, 1632 (2018).

12 Yann LeCun, Corinna Cortes & J.C., C. *The MNIST database of handwritten digits*, <http://yann.lecun.com/exdb/mnist/>

13 Shi, C., Dubois, M., Wang, Y. & Zhang, X. High-speed acoustic communication by multiplexing orbital angular momentum. *Proceedings of the National Academy of Sciences* **114**, 7250-7253 (2017).

14 Jiang, X., Liang, B., Cheng, J. C. & Qiu, C. W. Twisted Acoustics: Metasurface‐Enabled Multiplexing and Demultiplexing. *Advanced Materials* **30**, 1800257 (2018).

15 Wang, J. *et al.* Terabit free-space data transmission employing orbital angular momentum multiplexing. *Nature Photonics* **6**, 488 (2012).

16 Willner, A. E., Wang, J. & Huang, H. A Different Angle on Light Communications. *Science* **337**, 655 (2012).
